# Winter to winter recurrence of atmospheric circulation anomalies over East Asia and its impact on winter surface air temperature anomalies

**DOI:** 10.1371/journal.pone.0171641

**Published:** 2017-02-08

**Authors:** Xia Zhao, Guang Yang

**Affiliations:** 1 Key Laboratory of Ocean Circulation and Waves, and Institute of Oceanology, Chinese Academy of Sciences, Qingdao, China; 2 Laboratory for Ocean and Climate Dynamics, Qingdao National Laboratory for Marine Science and Technology, Qingdao, China; 3 Center for Ocean and Climate Research, First Institute of Oceanography, State Oceanic Administration, Qingdao, China; 4 Laboratory for Regional Oceanography and Numerical Modeling, Qingdao National Laboratory for Marine Science and Technology, Qingdao, China; University of Vigo, SPAIN

## Abstract

The persistence of atmospheric circulation anomalies over East Asia shows a winter to winter recurrence (WTWR) phenomenon. Seasonal variations in sea level pressure anomalies and surface wind anomalies display significantly different characteristics between WTWR and non-WTWR years. The WTWR years are characterized by the recurrence of both a strong (weak) anomalous Siberian High and an East Asian winter monsoon over two successive winters without persistence through the intervening summer. However, anomalies during the non-WTWR years have the opposite sign between the current and ensuing winters. The WTWR of circulation anomalies contributes to that of surface air temperature anomalies (SATAs), which is useful information for improving seasonal and interannual climate predictions over East Asia and China. In the positive (negative) WTWR years, SATAs are cooler (warmer) over East Asia in two successive winters, but the signs of the SATAs are opposite in the preceding and subsequent winters during the non-WTWR years.

## Introduction

A considerable amount of climatology research focuses on winter temperatures. Winter is the season most obviously affected by global warming; therefore, research on temperature change during the winter helps scientists better understand climate warming. Additionally, disastrous winter climates are closely related to temperature changes that influence people’s lives. Local large-scale atmospheric circulation anomalies are an important factor affecting the winter weather and climate over East Asia. They are closely related to the intensity of the East Asian winter monsoon, which is closely linked with the Siberian High [1–5]. Therefore, a better investigation of the characteristics of and physical mechanism for local atmospheric circulation anomalies should improve our understanding of the East Asian climate.

The persistence of atmospheric circulation anomalies has strong seasonal dependence over East Asia, which is called winter to winter recurrence (WTWR). WTWR was first discovered in mid-latitudinal sea surface temperature anomalies (SSTAs), which recur from one winter to the next with no persistence during the intervening summer [6–12]. Because this phenomenon effectively prolongs the memory of winter SSTAs for more than one year, a better understanding of WTWR may improve our ability to make seasonal climate predictions. In addition to SSTAs, anomalous atmospheric circulation in the mid–high latitudes of the Northern Hemisphere was also found to exhibit the WTWR phenomenon in the central North Pacific, East Asia, and the North Atlantic [13]. Furthermore, atmospheric WTWR in the central North Pacific was investigated by Zhao and Li [14–15], who focused on its interannual variability. They found that forcing of the atmospheric WTWR exerts an enormous influence on the oceanic WTWR in this region.

However, the interannual variability of the atmospheric WTWR over East Asia and its impact on winter temperatures remains unclear. Zhao and Li [13] analyzed the climatological features of the atmospheric WTWR in East Asia; its interannual variability has not drawn attention so far. In the present paper, discussing the interannual variability of the East Asian WTWR could familiarize us with the persistence of atmospheric circulation anomalies. Moreover, analysis of its impact on winter temperature anomalies is useful in improving seasonal and interannual climate predictions over East Asia and China.

The remainder of this manuscript is organized as follows. The datasets used are described in Section 2, and the WTWR of the atmospheric circulation anomalies over East Asia and the interannual variability of WTWR are examined in Section 3. Atmospheric circulation anomalies during WTWR and non-WTWR years are presented in Section 4. The impact of the WTWR on winter temperature anomalies is analyzed in Section 5. A summary and discussion are provided in Section 6.

## Data

The primary datasets used in the present paper are from the National Center for Environmental Prediction–National Centers for Atmospheric Research (NCEP–NCAR) reanalysis data [16]. Observed surface air temperature (SAT) data over China are derived from a 160-station monthly dataset for China provided by the China Meteorological Administration. The results reported in this paper are for the period between January 1951 and December 2014. The climatological annual cycle of each variable has been subtracted. In addition, the 44-year (1958–2001) European Center for Medium-Range Weather Forecasts (ERA-40) reanalysis [17] is also used to verify our results.

## Results

### WTWR of the atmospheric circulation anomalies over East Asia and the interannual variability of WTWR

**Fig 1:**
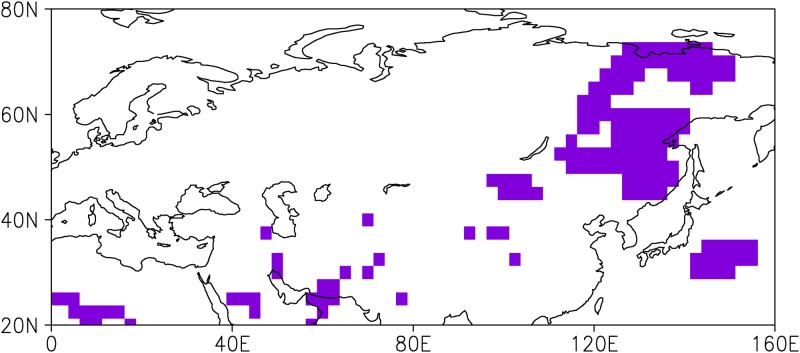
Geographical distribution of the WTWR of sea level pressure anomalies (SLPAs) in the Eurasian continent for the starting month of February.

**Fig 2:**
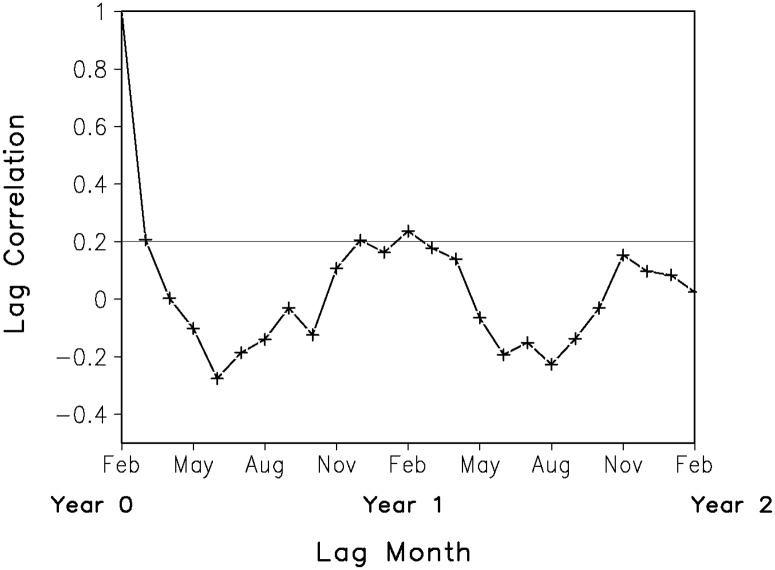
Lag correlations between the SLPAs in February and the SLPAs in each subsequent month over East Asia (45°N–75°N, 100°E–140°E). The thin solid line represents the 90% confidence level.

Let us first examine the geographical distribution of the WTWR of anomalous atmospheric circulation over East Asia. Lag correlation analysis is performed at each grid point of the sea level pressure anomalies (SLPAs), which obtains more accurate recurrence areas compared to the selection of regions or specific spatial patterns. At one grid point, there is a WTWR year if the lag correlation coefficient for the starting month of the winter season (February) drops to an insignificant level during summer and recurs during the following winter (January to March). This method was explained in more detail by Zhao and Li [13]. Fig 1 shows that the WTWR over the Eurasian continent is mainly located in East Asia. The WTWR region is defined as a box bounded by 45°N–75°N, 100°E–140°E. Then, the lag correlations of the SLPAs in this WTWR region based on a starting month of February are calculated (Fig 2). The persistence of SLPAs starting in the first winter (February) decreases markedly during the summer (June to August) and increases in the second winter, with a maximum from November to April.

The above lag correlation was used in previous studies to define the SSTAs WTWR [9]. Thus, from this original definition of WTWR (Fig 2), we determine the existence of WTWR over East Asia in each year to study its interannual variability. A positive (negative) WTWR year is chosen if positive (negative) SLPAs during the first winter (January to March) are larger (smaller) than anomalies during the following summer (June to August) and anomalies during the second winter (January to March) are larger (smaller) than those in the summer before and have the same sign as those in the first winter. Then, the remaining years are categorized into positive and negative non-WTWR years. Finally, we identified 35 WTWR years (positive cases: 1951, 1952, 1953, 1954, 1955, 1956, 1957, 1960, 1963, 1964, 1965, 1969, 1970, 1971, 1984; negative cases: 1967, 1975, 1978, 1981, 1982, 1986, 1987, 1988, 1989, 1992, 1995, 1996, 1997, 1998, 2001, 2002, 2003, 2006, 2007, 2008) and 28 non-WTWR years (positive cases: 1958, 1961, 1966, 1972, 1974, 1977, 1980, 1985, 1991, 1994, 2000, 2005, 2010, 2012; negative cases: 1959, 1962, 1968, 1973, 1976, 1979, 1983, 1990, 1993, 1999, 2004, 2009, 2011, 2013) in the WTWR region over East Asia during the period 1951–2014.

### Atmospheric circulation anomalies during WTWR and non-WTWR years

**Fig 3:**
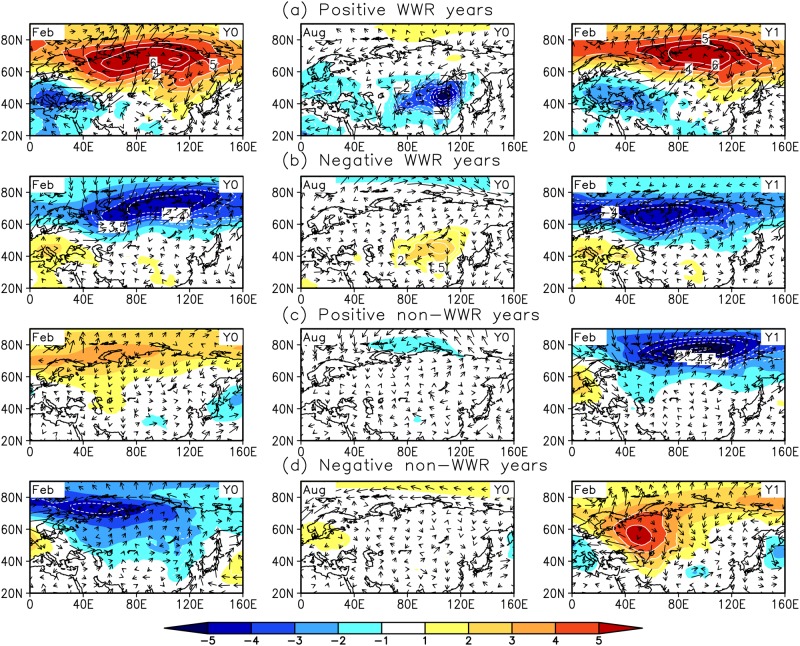
The composite SLPAs and surface wind anomalies. **(a)** The positive WTWR cases, (b) the negative WTWR cases, (c) the positive non-WTWR cases and (d) the negative non-WTWR cases. Y0 and Y1 represent the current year and the next year, respectively. The contours indicate that the anomalies are significant at the 90% confidence level according to Student’s t test.

The characteristic patterns of atmospheric circulation anomalies in WTWR and non-WTWR years are examined through composite analysis. Fig 3a and 3b give the temporal evolution for the horizontal structure of the composite SLPAs and surface wind anomalies over the Eurasian continent during the positive and negative WTWR events, respectively. The climatological annual cycle of each variable was subtracted from the monthly variables before composite analysis. The large-scale atmospheric circulation anomalies in Eurasia show significant seasonal variation during the WTWR situations. For the positive situations (Fig 3a), during the first winter (February), large areas of positive SLPAs cover the mid-high latitudes, centered over the Eurasian continent. Meanwhile, anticyclonic wind anomalies prevail over Eurasia. During summer (September), the SLPAs change to a weak low pressure, accompanied by cyclonic surface wind anomalies. During the second winter (February), the atmospheric circulation anomalies occur again, and the geographical distribution and strength of the anomalous high pressure center and anticyclone resemble those in the first winter. Fig 3b shows the situations during the negative WTWR events. The pattern of atmospheric circulation anomalies over the Eurasian continent is the reverse of the positive situations. A corresponding characteristic is the reappearance of anomalous low pressure and a cyclone in the Eurasian continent in two successive winters with no persistence in summer.

Fig 3c and 3d show the characteristics of the atmospheric circulation anomalies during the non-WTWR events. For the first winter, the anomalies over Eurasia have features similar to that during WTWR events. For the positive (negative) cases, the atmospheric state shows high (low) pressure anomalies accompanied by an anomalous anticyclone (cyclone) over the Eurasian continent. Then, the anomalies become weak during the summer. Obviously, significant differences appear in the second winter. Unlike in WTWR event conditions, atmospheric circulation continues to weaken or becomes the opposite sign during the second winter. Thus, the atmospheric circulation anomalies over Eurasia do not recur in non-WTWR cases.

Therefore, the seasonal variations in atmospheric circulation anomalies over Eurasia have significant differences between WTWR and non-WTWR events. Generally, a WTWR event is characterized by the recurrence of the anomalous Siberian High and East Asian winter monsoon (EAWM). The Siberian High strengthens (weakens) in the first winter of the positive (negative) cases, which is accompanied by a strengthened (weakened) winter monsoon in East Asia. The state is the same in the second winter. For the non-WTWR events, the Siberian High also strengthens (weakens) in the first winter of the positive (negative) cases. In the second winter, however, the Siberian High weakens (strengthens) and is accompanied by a weakened (strengthened) winter monsoon in East Asia. During winter, atmospheric circulation anomalies are controlled by the EAWM. Obviously, WTWR of anomalous atmospheric circulation is important to the winter climate in East Asia.

### Impact on surface air temperature anomalies

**Fig 4:**
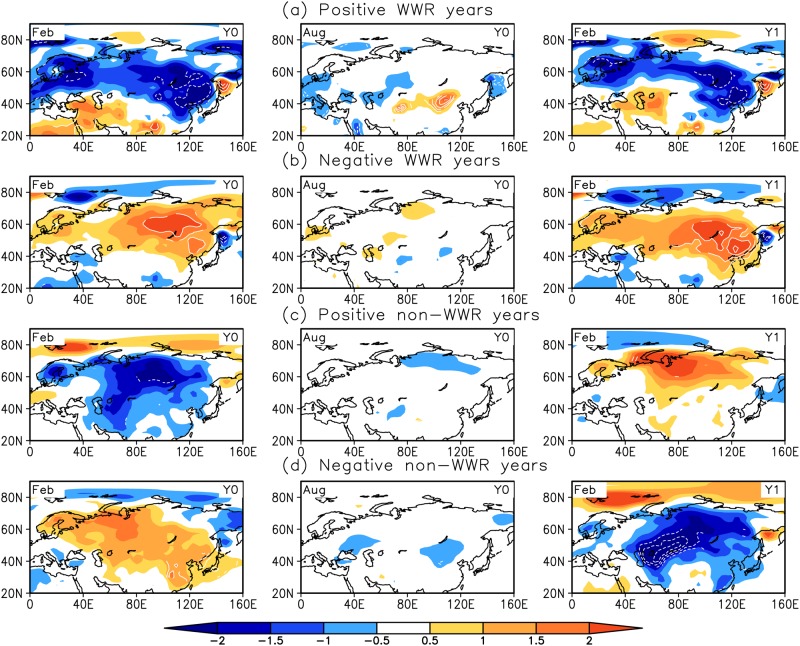
Same as in Fig 3, but for the SATAs.

Meridional wind is a crucial aspect of the EAWM [18–21] because it carries cold air outbreaks southward. A strong winter monsoon corresponds to cold temperatures and a weak winter monsoon corresponds to warm temperatures. The WTWR of anomalous atmospheric circulation will inevitably impact air temperatures over East Asia from one winter to the next. Fig 4 gives the temporal evolution of the composite SAT anomalies (SATAs) during WTWR and non-WTWR events. In the positive (negative) WTWR state, during two successive winters, the SATAs are cooler (warmer) over most of the Eurasian continent, showing more pronounced anomalies over East Asia. During the intervening summer, the SATAs are relatively weaker. For the non-WTWR events, the signs of the SATAs are opposite over East Asia in the preceding and following winters.

**Fig 5:**
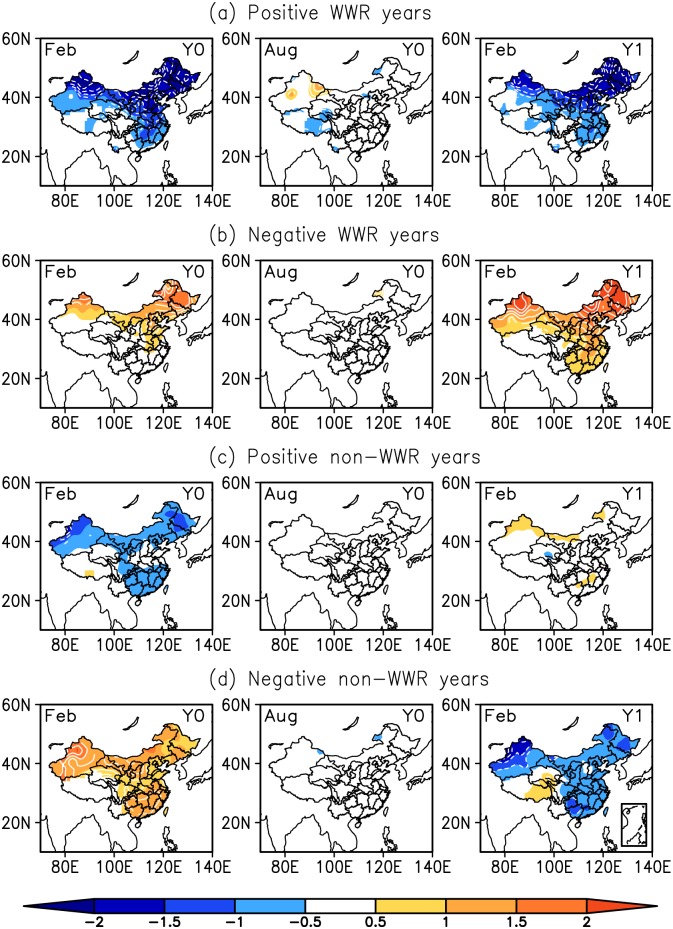
Same as in Fig 4, but for the SATAs from the 160-station monthly dataset for China.

For WTWR and non-WTWR events, SATAs also show characteristic differences over China (Fig 5). During the WTWR events, the spatial pattern of the SATAs shows homogeneity in the current and ensuing winters, which indicates that the SATAs in China have the same variability sign during two successive winters. This homogeneous pattern also appeared in other papers [22–23]. Moreover, their results reflected a warming trend over China during the period 1951–2014, relatively colder conditions before the 1980s and warmer conditions after the 1980s. Similarly, our result described in Section 3 also suggests the interdecadal variability of the atmospheric WTWR over East Asia. Most of the positive WTWR years appear before the 1980s, which corresponds to cold temperature anomalies in winter, and most of the negative WTWR years exist after the 1980s, which corresponds to warm temperature anomalies in winter.

**Fig 6:**
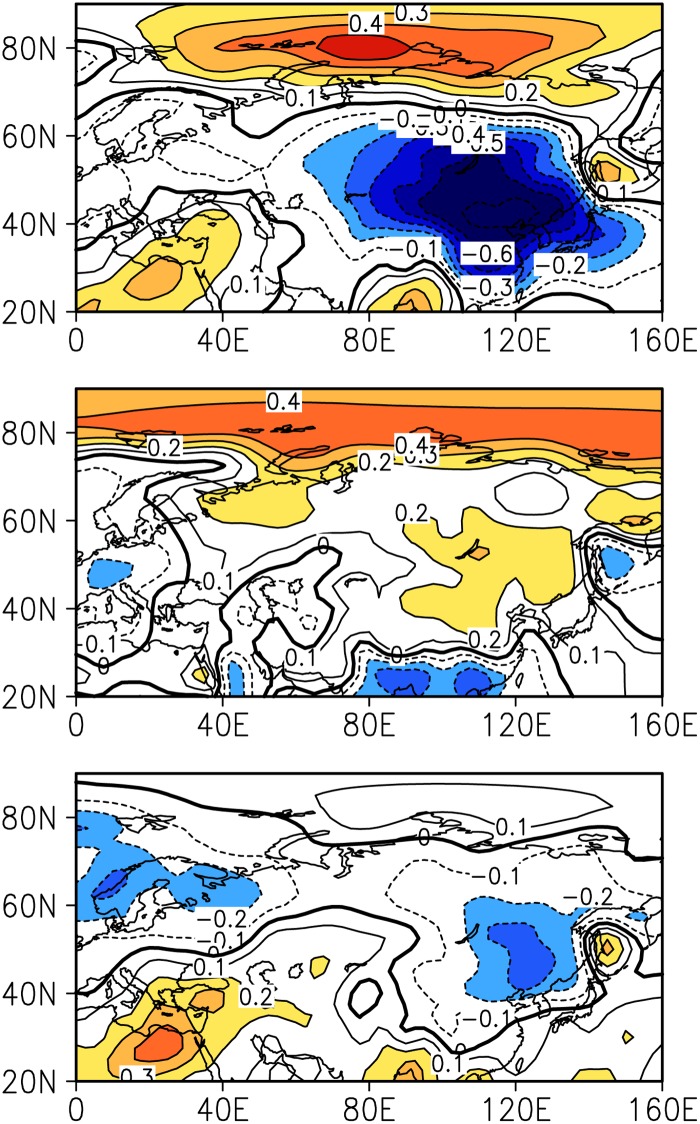
Correlation between SLPAs and SATAs over East Asia. The correlations among the SLPAs in the winter and SATAs in the winter of the current year (upper panel), intervening summer (middle panel) and in the winter of the ensuring year (lower panel) of WTWR years. The shading indicates positive and negative correlations above the 90% significance level.

The WTWR of circulation anomalies is associated with that of SATAs over East Asia, implying that the former is possibly an important hint for seasonal and interannual predictions of the latter. Fig 6 gives the correlation coefficients between circulation anomalies, and the SATAs over East Asia in the current year and the ensuring year of WTWR years are calculated. SLPAs in the winter and SATAs in the winter of the current year and the following year have significant negative correlation coefficients over East Asia, but the correlation coefficients are positive in the intervening summer. Furthermore, using the relationship between the SLPAs in the current winter and the SATAs in the following winter over East Asia, we fit a linear regression equation and use variance analysis to test the validity of the equation. The regression equation is finally fitted in the form given by

SATAs(t+1)=−0.23×SLPAs(t)+0.01

where SATAs(t+1) are the SATAs in the winter of the *(t+1)*th year (t = 1, …, 63) over the period of 1952–2015, and SLPAs(t) are the SLPAs in the winter of the *t*th year. Because the value of the *F*-test is 11.1, exceeding the significant value of 7.1 at the 0.01 level, the equation is statistically significant. Therefore, SLPAs(t) can be considered a better predictor of SATAs(t+1).

## Summary and discussion

Zhao and Li [13] discussed the mean climatic characteristics of the WTWR of atmospheric circulation over East Asia. The present study investigated its interannual variability. During the period 1951–2014, 35 WTWR years and 28 non-WTWR years are identified in the WTWR region over East Asia. The seasonal variations of the SLPAs and the surface wind anomalies show significant differences between the WTWR and non-WTWR events. On the whole, the WTWR over East Asia is characterized by the recurrence of the anomalous Siberian High and EAWM during two successive winters. The Siberian High strengthens (weakens) in the first winter of the positive (negative) cases, which is accompanied by a strengthened (weakened) winter monsoon in East Asia. The state is the same in the second winter. During the non-WTWR events, the Siberian High also strengthens (weakens) in the first winter of the positive (negative) cases. However, in the second winter, the signs of the Siberian High and winter monsoon are reversed.

Furthermore, the WTWR of the atmospheric circulation anomalies will inevitably impact the air temperatures over East Asia and China from one winter to the next. In the positive (negative) WTWR cases, the SATAs are cooler (warmer) over East Asia in two successive winters, which are relatively weak during the intervening summer. However, for the non-WTWR events, the signs of the SATAs are opposite in the preceding and following winters. Thus, the WTWR of anomalous atmospheric circulation contributes to the recurrence of winter air temperature anomalies over East Asia, which is useful information for improving seasonal and interannual climate prediction in East Asia and China.

Due to the large meridional extent of the EAWM, the climate anomalies associated with the EAWM are not consistent between the mid-high latitudes and the low latitudes of East Asia. Several studies have revealed the presence of two distinct modes associated with the EAWM variability. Kang et al. [24–25] first identified two leading modes of wintertime surface air temperature in China. Wang et al. [26] extracted a northern mode and a southern mode of EAWM variability based on wintertime surface air temperature anomalies over Asia and the western Pacific. Liu et al. [20] and Chen et al. [21, 27] derived the two modes of the EAWM based on surface wind anomalies. Our analysis indicated that the WTWR over East Asia is confined to the north of 40°N (Fig 1), and its impact on winter SATAs shows a contrast between the north and the south over East Asia (Figs 3 and 4). This result further suggests the inconsistencies in the East Asian winter climate and the associated EAWM between the mid-high latitudes and the low latitudes.

There are studies indicating the discrepancy between the different reanalysis datasets [28–29]. Therefore, we repeat the analysis using the ERA-40 reanalysis datasets (figures not shown). Although some differences can be detected in the two different reanalysis datasets, the results obtained from the ERA-40 reanalysis data, including the geographical distribution of the WTWR of SLPAs in the Eurasian continent, the persistence characteristic of the winter SLPAs over East Asia, the seasonal variations in the SLPAs, the surface wind and the SATAs between WTWR and non-WTWR years, basically agree with those from the NCEP–NCAR reanalysis datasets. The results in the present study are not strongly dependent on the reanalysis data.

Previous studies have shown that the EAWM experienced a decadal change around the mid-1980s [24, 30] and mainly occurred in the northern part of East Asia [31], which leads to an obvious increase in the winter SATAs over large areas of the East Asia [24, 32]. Similarly, our results show that atmospheric WTWR in East Asia also has interdecadal variability. Most of the positive WTWR years appeared before the 1980s, and most of the negative cases appeared after the 1980s (Section 3). This result indicates that since the 1980s, the Siberian High weakens in two successive winters, which is accompanied by a weakened EAWM and warm SATAs in China. Further discussing the interdecadal variability of the atmospheric WTWR in East Asia and its climatic impact is worthwhile.

Zhao and Li [15] have investigated the dynamics responsible for atmospheric WTWR over the North Pacific. However, the causes of the WTWR of atmospheric circulation anomalies over East Asia are still unknown. What are the roles of the internal atmospheric dynamics in atmospheric WTWR? Is the influence of external forcing, e.g., sea surface temperature anomalies, significant or not? We find that when atmospheric WTWR appears over East Asia, the anomalous Aleutian Low in the North Pacific also recurs in two successive winters. Zhao and Li [14] indicated that the North Pacific WTWR is a phenomenon of the entire air-sea system. Thus, further investigation of the relationship between the atmospheric WTWR over East Asia and the air-sea WTWR in the North Pacific is required.
